# Refractory Hypokalemia of Pregnancy: A Rare Case of Non-Aldosterone Mediated Hypokalemia

**DOI:** 10.7759/cureus.79242

**Published:** 2025-02-18

**Authors:** Deekshita Valiveti, Olivia Lahey, Karim Nooruddin, Brandi Addison

**Affiliations:** 1 Internal Medicine, Corpus Christi Medical Center Bay Area, Corpus Christi, USA

**Keywords:** aldosterone renin ratio, geller syndrome, refractory hypokalemia, serum cortisol, severe hypokalemia etiologies

## Abstract

There is a wide differential for a patient presenting with hypokalemia and hypertension in pregnancy. Of these, Geller syndrome is a rare variant of mineralocorticoid receptor that leads to concomitant hypokalemia and gestational hypertension. Progesterone has been shown to have a high affinity for the mineralocorticoid receptor and thus antagonizes aldosterone functioning. However, in Geller syndrome, there is a mutation of the mineralocorticoid receptor with a resultant gain of function. Activation of the mutated receptor is characterized by hypertension and hypokalemia, which is exacerbated by the effect of progesterone and thereby presenting during pregnancy. Genetic testing can confirm the diagnosis of Geller syndrome. The management is supportive therapy and requires close monitoring of the patient and her fetus. Delivery of the fetus results in the resolution of both hypertension and hypokalemia. This report describes the case of a 25-year-old female patient with a history of alpha-1 antitrypsin deficiency who presented with symptomatic hypokalemia refractory to treatment in her third trimester.

## Introduction

Hypokalemia has a variety of etiologies including loss through the gastrointestinal system, renal excretion, intracellular shift, and rarely decreased intake. Hypokalemia in pregnancy can be seen in Geller syndrome, a rare variant of mineralocorticoid receptor that leads to concomitant hypokalemia and gestational hypertension [1]. 

Since being first described by Geller et al. in 2000 [1], there have been roughly 10 reported cases [2-10]. Genetic testing confirms the diagnosis of Geller syndrome. The management is supportive therapy and requires close monitoring of the patient and her fetus. Delivery of the fetus results in the resolution of both hypertension and hypokalemia [1]. 

This case report of a 25-year-old pregnant woman with refractory hypokalemia emphasizes the importance of swift and accurate diagnosis and treatment.

## Case presentation

A 25-year-old female patient, G4 P0120 (23rd-week perinatal death), presented at 29 weeks gestation for consultation regarding severe hypokalemia with paresthesias, pedal edema, lethargy, and generalized weakness.

The patient recalled that she has had issues with low potassium during the current and prior pregnancies. In previous pregnancies, she required oral potassium (which she was unable to tolerate) and thus necessitated frequent infusions of potassium in the emergency room. Once the patient was postpartum, she was able to maintain her serum potassium levels with dietary management. During the current pregnancy, her prenatal course was rather uncomplicated with the exception of nausea and vomiting (one to two episodes daily). She also endorsed palpitations. The patient denied any worsening gastrointestinal symptoms such as increased vomiting or diarrhea that could explain the persistent hypokalemia. She also denied any recent febrile illness. Also during the current pregnancy, she presented to the emergency department on seven separate occasions for elevated blood pressure and was noted to have low potassium levels requiring intravenous (IV) supplementation. Aside from hypokalemia, her remaining outpatient laboratory testing and sonograms were unremarkable. Her home medications included: albuterol, fluticasone propionate/salmeterol, gabapentin, and prenatal vitamins. She specifically denied taking over-the-counter medications, IV or recreational drug use, and tobacco or alcohol use.

Vital signs on admission showed blood pressure 112/56 mmHg, heart rate 87 beats/minute, respiratory 17 breaths/minute, oxygen saturation (SpO2) 95% on room air, temperature 97.9 F. Physical examination was notable for trace pedal edema and gravid uterus consistent with 29 weeks gestation. On laboratory evaluation, she was found to have significant hypokalemia of 2.7 mEq/L and was admitted to the inpatient antepartum unit for further workup and treatment. Labs on admission can be found in Table 1.

**Table 1:** Urine studies and serum chemistry obtained at admission

Test	Result	Reference value		
Urine creatinine	31.59 mg/dL	No established reference range		
Urine chloride	119 mmol/L			
Urine potassium	22 mmol/L			
Urine sodium	102 mmol/L			
24-hour urine protein	320 mg/24 hours	0-165 mg/24 hours		
Renin	2.707 ng/mL/hour	0.167-5.380 ng/mL/hour		
Aldosterone	<1.0 ng/dL	0.0-30.0 ng/dL		
Repeat renin	0.757 ng/mL/hour	0.167-5.380 ng/mL/hour		
Repeat aldosterone	<1.0 ng/dL	0.0-30.0 ng/dL		
Sodium	139 mmol/L	133-145 mmol/L		
Potassium	2.7 mmol/L	3.6-5.2 mmol/L		
Chloride	106 mmol/L	100-108 mmol/L		
Carbon dioxide	25 mmol/L	22-32 mmol/L		
Glucose	73 mg/dL	65-99 mg/dL		
Blood urea nitrogen	4 mg/dL	6-20 mg/dL		
Creatinine	0.64 mg/dL	0.60-1.00 mg/dL		
Glomerular filtration rate	126 mL/min	71-165 mL/min		
Total protein	5.6 g/dL	6.4-8.2 g/dL		
Albumin	2.1 g/dL	3.4-5.0 g/dL		
Globulin	3.5 g/dL	1.5-3.8 g/dL		
Calcium	7.8 mg/dL	8.7-10.5 mg/dL		
Total bilirubin	0.1 mg/dL	0.0-1.0 mg/dL		
Aspartate aminotransferase (AST)	28 units/L	15-37 units/L		
Alanine aminotransferase (ALT)	12 units/L	30-65 units/L		
Alkaline phosphatase (ALP)	140 units/L	50-136 units/L		
Magnesium	1.9 mg/dL	1.8-2.4 mg/dL		

After being started on vigorous IV potassium supplementation, her symptoms largely resolved and intermittent palpitations dissipated. A review of her records revealed that her fractional excretion of urinary potassium was 12.6% in the presence of hypokalemia and replacement of potassium at 90 mEq/day. This indicates potassium wasting by the kidneys. Her magnesium level was 1.5 mg/dL which was repleted daily. The patient was not known to have any history of renal dysfunction. Nephrology was thus consulted and believed that there could be an association with pregnancy-induced hypokalemia.

Further workup revealed normal thyroid-stimulating hormone (TSH) and cortisol levels, with a renin level of 0.757 ng/mL/hour (normal range: 0.167-5.380 ng/mL/hour) and suppressed aldosterone level (below 1 ng/dL) (Table 2). Given the fractional excretion of potassium at 12.6% and simultaneous suppression of aldosterone, this incongruence implies renal potassium wasting [2-5,11].

**Table 2:** Quantity of potassium supplementation during hospitalization KCl: potassium chloride; D5W: dextrose 5% in water; LR: lactated Ringer's solution

Hospital day and time	Potassium administered
Day 1 0600	Oral KCl 30 mEq
Day 1 0700	Intravenous D5W + KCl @ 100 cc/hour
Day 1 1200	Intravenous D5-LR + KCl @ 125 cc/hour
Day 1 1200	Oral KCl 30 mEq
Day 1 1700	Oral 30 mEq
Day 1 2100	Intravenous D5-LR + KCl @ 125 cc/hour
Day 2 0800	Oral KCl 30 mEq
Day 2 1200	Oral KCl 30 mEq
Day 2 1800	Oral KCl 30 mEq
Day 3 0800	Oral KCl 30 mEq
Day 3 1300	Oral KCl 40 mEq
Day 3 1700	Oral KCl 40 mEq

Geller syndrome is a rare disease that manifests during pregnancy and causes clinical findings of severe hypokalemia and gestational hypertension. Normalization of blood pressure and potassium following delivery strongly suggests Geller syndrome. The diagnosis is further supported by low serum aldosterone and plasma renin in addition to hypokalemia despite an elevated urinary potassium-creatinine ratio. The presence of hypertension in addition to hypokalemia, despite renal-potassium wasting, implies a hyperaldosteronism-like state. However, suppressed serum aldosterone indicates the diagnosis of an activating mineralocorticoid receptor mutation [4]. Diagnosis is suggested by clinical findings and can be confirmed with genetic testing [1].

**Figure 1:**
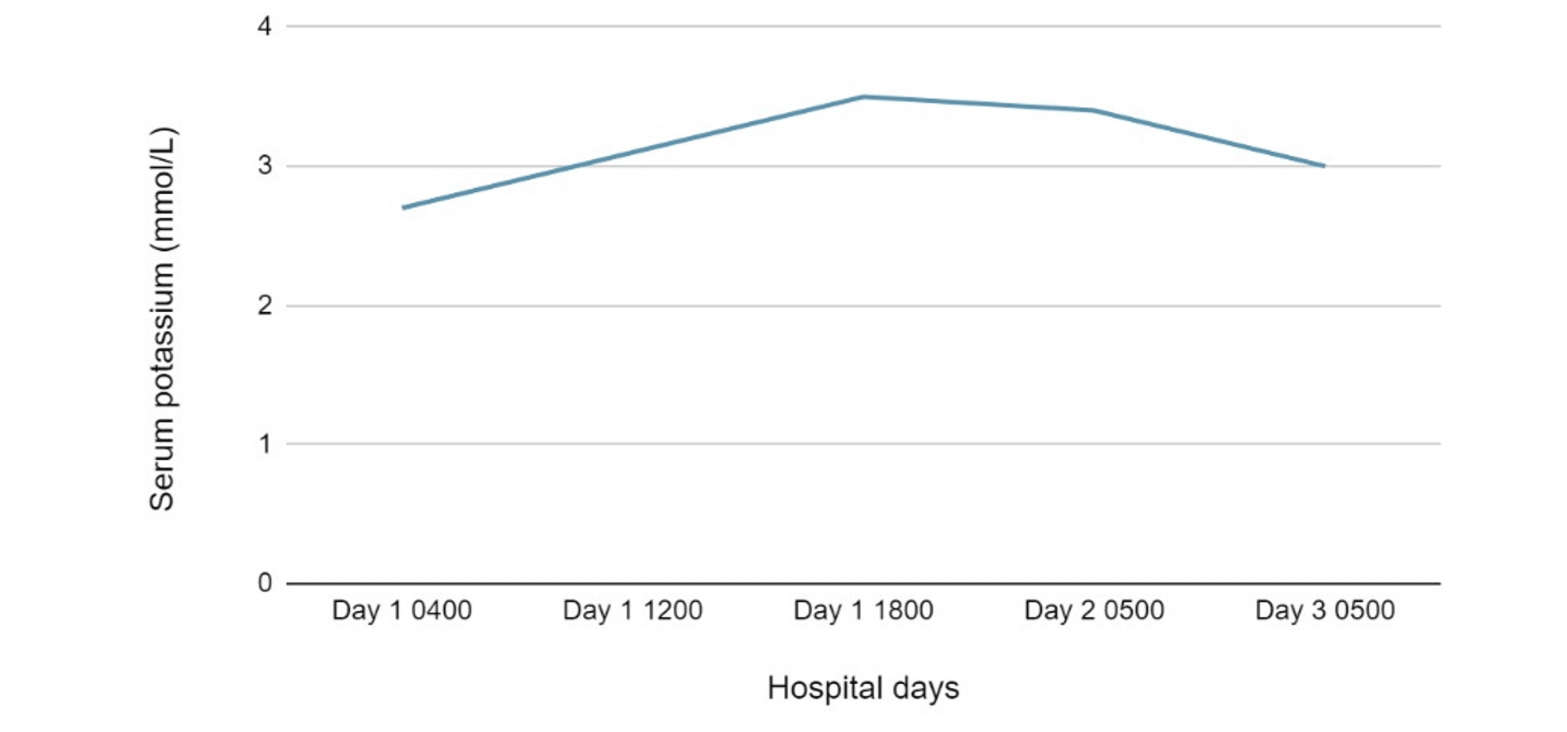
Serum potassium trend during hospitalization

Our patient's findings of recurrent pregnancy with severe hypokalemia, gestational hypertension, suppressed renin and aldosterone, and elevated urine potassium-creatinine ratio support the diagnosis of Geller syndrome. Following aggressive intravenous potassium supplementation (Figure 1), the patient clinically improved and she was discharged home with oral potassium chloride 40 mEq three times daily and magnesium oxide 400 mg twice daily. Note that there was a discussion on whether or not to initiate amiloride; however, the patient was planning to travel cross-country to return home and, as close follow-up was undetermined, she was strongly advised to discuss initiation of amiloride with her maternal-fetal medicine physician once she returned home.

## Discussion

The differential diagnosis of a patient presenting with hypokalemia is broad, including genetic conditions such as congenital adrenal hyperplasia (CAH), acquired conditions such as mineralocorticoid excess, decreased potassium intake, or increased potassium losses. Liddle syndrome was also a consideration in this patient; however, we would have expected to see early-onset hypertension and hypokalemia. Moreover, refractory hypokalemia can be seen in Gitelman and Bartter syndrome; however, normal or low blood pressure with elevated renin and aldosterone levels is expected in patients who are diagnosed with these syndromes [6,12,13]. Although our patient exhibited a lack of hypertension on this admission, she did note mild gestational hypertension in previous pregnancies.

More recently, other etiologies for hypokalemia have been studied. Notably, Geller et al., in 2000, identified pregnant individuals presenting with gestational hypertension and hypokalemia [1]. The proposed mechanism is caused by an agonist effect of the excessive production of progesterone and its action on a mutated mineralocorticoid receptor, leading to severe hypokalemia and gestational hypertension [1,6]. As seen in our patient, renin and aldosterone levels are usually suppressed in this syndrome[2-5,11]. Genetic testing remains the gold standard for confirmation of the diagnosis [2]. However, due to limitations, this was not performed in our patient for confirmation of the presumed Geller syndrome diagnosis. 

Treatment is generally supportive and focuses on managing complications of gestational hypertension and hypokalemia which usually resolve after delivery [14]. The current literature suggests that some patients have responded well to amiloride, while spironolactone has been noted to worsen blood pressure by activating the mutated mineralocorticoid receptor, therefore contraindicated in Geller syndrome and also in pregnancy [15].

## Conclusions

Given the novelty and rarity of the condition, both the early recognition and awareness of Geller syndrome are crucial to avoid complications for the patient and fetus from hypokalemia and hypertension. Clinicians should be vigilant when evaluating recurrent hypokalemia of pregnancy. Early identification and management can mitigate hospitalizations for severe hypokalemia and the development of periodic paralysis. Clinicians should also focus on patient education regarding high chances of recurrence during future pregnancies. Finally, consistent follow-up for monitoring serum potassium levels in addition to blood pressure is necessary for the treatment of Geller syndrome and other syndromes of apparent mineralocorticoid excess. Although benefit from amiloride has been suggested, future studies can focus on its role in treatment and explore potential teratogenic effects.
